# Magnitude and Associated Factors of Perceived Stigma among Adults with Mental Illness in Ethiopia

**DOI:** 10.1155/2019/8427561

**Published:** 2019-03-27

**Authors:** Etsedingl Hadera, Endalamaw Salelew, Eshetu Girma, Sandra Dehning, Kristina Adorjan, Markos Tesfaye

**Affiliations:** ^1^Mekelle University Ayder Comprehensive Specialized Hospital, Mekelle, Ethiopia; ^2^Department of Psychiatry College of Medicine and Health Science University of Gondar, Gondar, Ethiopia; ^3^Department of Preventive Medicine, School of Public Health, Addis Ababa University, Ethiopia; ^4^Department of Child and Adolescent Psychiatry, Ludwig Maximilian University Munich, Munich, Germany; ^5^Center for International Health, Ludwig Maximilian University Munich, Munich, Germany; ^6^Department of Psychiatry and Psychotherapy, Ludwig Maximilian University Munich, Munich, Germany; ^7^Institute of Psychiatric Phenomics and Genomics (IPPG), University Hospital, LMU Munich, Germany; ^8^St. Paul's Hospital Millennium Medical College, Addis Ababa, Ethiopia

## Abstract

**Background:**

Many people with mental illness perceive and experience stigma caused by other people's knowledge, attitudes, and behavior. The stigma can lead to patients' impoverishment, social marginalization, poor adherence to medication, and low quality of life, worsen the disease, decrease health-seeking behavior, and have a negative impact on socioeconomic well-being. Therefore, this study aimed to explore these issues.

**Objective:**

To assess the magnitude and associated factors of perceived stigma among adults with mental illness in an Ethiopian setting.

**Methods:**

A facility-based, cross-sectional study design with a consecutive sampling technique was employed from September 1 to 30, 2012. Data for perceived stigma were assessed by using the perceived devaluation-discrimination (PDD) scale from new or returning patients. The data was analyzed by using the Statistical Package for the Social Sciences (SPSS) version 20. The results were described with the frequency table, graph, mean, and standard deviation. Bivariate analysis was used to get candidate variables for multivariate logistic regression analysis. Variables with a* P* value of < 0.05 at multivariate analysis were considered statistically associated with perceived stigma.

**Results:**

A total of 384 participants were interviewed and the response rate was 100%. The prevalence of high and low perceived stigma was 51% and 44%, respectively. Having substance use history (AOR=0.6, 95% CI: 0.4–0.9) and family support (AOR=2.5, 95% CI: 1.5–4.3) and medication side effects (AOR=0.6, 95% CI: 0.5–0.8) were associated statistically with higher perceived stigma of people with mental illness.

**Conclusion:**

Perceived stigma is a major problem of adults with mental illness in this outpatient setting in Ethiopia. Patients who had substance use and family support and medication side effects were more likely to have high perceived stigma. Therefore, screening and management of substance use, social support, and medication side effect should be strengthened for people with mental illness.

## 1. Introduction

Stigma refers to attitudes and beliefs that lead people to reject, avoid, or fear those they perceive as being different [1]. Perceived stigma is fear of being discriminated against or the fear of enacted stigma and arises from society's belief [2]. According to World Health Organization (WHO) Report 2010, people with mental and psychosocial problems are subjected to high levels of stigma and discrimination because of widely held misconceptions about the causes and nature of mental health conditions [3]. The stigma of mental illness is a severe burden for people with mental illness in both their private and public lives and also affects their relatives [4]. In the United Kingdom, nearly nine out of ten people (87%) with mental health problems have been affected by stigma and discrimination, and 73% of affected people say they have stopped doing things they wanted to do due to fear of stigma and discrimination [5]. Studies in Pakistan indicated that perceived stigma is higher among persons with mental illness than diabetic patients [6]. Stigma toward mental illness is one of the leading reasons individuals with mental illness do not seek treatment for their conditions [7]. While effective treatment for mental disorders is available, barriers such as stigma against people with mental disorders prevent them from accessing and receiving help they need to stay well [8]. In 2001, WHO declared stigma to be the single most important barrier to overcome in the community [9]. Studies done in 16 countries of the world showed that 13.5% of the overall sample had perceived stigma (22.1% in developing countries and 11.7% in developed ones) [10]. In Germany studies showed that most people with mental illness expect negative reactions from the environment and patients with schizophrenia or depression who live in a small town perceive stigmatization more frequently than patients living in the city [11].

Patients with schizophrenia have more perceived stigma and treatment nonadherence [12]. A study in Singapore showed that 73% of people with schizophrenia had difficulty finding a job and 51% of them thought that neighbors and colleagues would neglect them due to their illness [13]. In Southern Poland people with mental illness living in a city and older patients perceived more discrimination in interpersonal relationships and employment [14]. A study performed in the United States found that perceived stigma was higher among males, those with lower socioeconomic status, and those without any family members or friends who had used mental health services [15].

Few studies were performed in African countries and among psychiatry patients in Nigeria high self-stigma was 21.6% [16] and in Ghana perceived stigma was 66.11% [17]. In studies in Ethiopia on schizophrenia patients perceived stigma was 83.5% [18]. Another study performed in Ethiopia among 212 individuals with a diagnosis of schizophrenia found that moderate to high internalized stigma was 46.7% [19].

WHO discussion paper 2009 showed that 66 million people worldwide have depression, 85% of whom live in low- and middle-income countries; 24 million people have an alcohol-related problem, 82% of whom live in low- and middle-income countries; and one million people commit suicide each year, 84% of whom were in low- and middle-income countries [20]. In sub-Saharan Africa, unipolar depression was the third leading cause of disease burden, and by the year 2020 it is expected to become the second leading cause of disease burden worldwide [21]. The overall prevalence of mental illness in South Africa was 25% among adults [22].

Perceived stigma among people with mental illness in Ethiopia has not been well studied. The objective of this study was to assess the magnitude and factors associated with perceived stigma among adults with mental illness attending the Outpatient Department of the Psychiatry Clinic at Jimma University Specialized Hospital (JUSH).

## 2. Methods and Materials

The study was conducted at the JUSH Department of Psychiatry, located in Jimma town 354 km southwest of Addis Ababa, the capital city of Ethiopia. JUSH is a teaching and tertiary level hospital and provides health services for more than 1.015 million people living in southwest Ethiopia. There were around 5405 follow-up psychiatry patients in the Psychiatry Clinic. The study was conducted from September 1 to 30, 2012.

A facility-based cross-sectional study design was employed. A single population proportion formula (with a 5% margin of error, 95% confidence level, and 50% proportion) was used to calculate sample size, found to be 384, and new patients who would come were included in the study so a correction formula was not used because the total population (N) was unpredictable. The total number of patients who were on follow-up care for the last 12 months was taken from patient records and then the average number of patients per day was calculated. All eligible persons coming to the outpatient clinic of age greater than or equal to 18 years were recruited by using a consecutive sampling technique until the required numbers of participants were obtained. Individuals who were actively psychotic, had no insight, and were unable to speak and hear were excluded from the study.

### 2.1. Instruments

A structured questionnaire was developed in the English language and translated into Amharic and Afaan Oromo and back to English by language professions who are native speakers. The questionnaire consisted of three parts that assessed patient sociodemographic variables, psychosocial factors, and perceived stigma and discrimination factors. Perceived stigma was assessed by using the perceived devaluation-discrimination (PDD) scale. The PDD scale is a 12-item tool which measures the extent to which a person believes that most people will devalue or discriminate against someone with a mental illness. PDD was measured on a 4-point Likert scale with possible scores ranging from 1 to 4 on the agreement scale (1 = strongly disagree, 2 = disagree, 3 = agree, and 4 = strongly agree). A high level of perceived devaluation and discrimination is indicated by agreement with six of the items and disagreement with six others. Items 1, 2, 3, 4, 8, and 10 were scored in reverse direction. The prevalence of high perceived stigma was defined as an item mean score of 2.5 or higher on the mean aggregated scale score (this criterion represented the “midpoint” on the 1–4-item scale) on PDD scales. Then perceived stigma scores were dichotomized as those participants scoring greater than or equal to the mean score of 2.5 on PDD scales as having “high perceived stigma” and those scoring below the mean score as having “low perceived stigma” [23]. This scale has been widely used across the world including Africa [17]. Previous work with this measure has shown that the PDD scale has internal consistency of *α* = 0.86 [23].

### 2.2. Data Collection Method

The data was collected by interviewing all psychiatric outpatients seeking treatment at the JUTH Psychiatric Clinic by four nurses with B.S. in psychiatry. The one professional with a master's in psychiatry was the supervisor and the principal investigator also participated in the supervision. For those data collectors and the supervisor one-day training was given. During the training the objective of the study was discussed. The data collection methods, tools, and how to handle ethical issues were discussed with the data collectors. The structured questionnaire was also discussed in detail on going through each question with clarification for doubt. The data-extraction form was designed to collect data from patient medical records on the number and types of diagnoses, duration of illness and treatment, side effects, and comorbid medical conditions.

### 2.3. Data Quality Control

A pretest was conducted (5% of sample size) before the main study to identify potential problems in the proposed procedures, such as administration of data collection tools, and to check the performance of the data collectors, and the data were excluded from the main data analysis. Regular supervision and control as well as support of data collectors by the supervisor and principal investigator were made daily and each completed questionnaire was checked and the necessary feedback was offered to interviewers the following morning. The collected data was properly reviewed and checked for completeness and consistency by the supervisor and principal investigator daily.

### 2.4. Data Analysis and Interpretation

After all necessary data were obtained, data was checked for completeness, edited, and entered into and cleaned with SPSS version 20.00. Data was explored to detect inconsistencies, outliers, and missing values. Numeric variables were summarized as mean, median, range, and standard deviation and categorical variables as frequency tables and bar graphs. A bivariate analysis was conducted to check the crude association between dependent and independent variables. Variables with a *P* ≤ 0.25 in the bivariate analysis were candidates for the multiple logistic regression analysis. Variables with a* P* value < 0.05 in the multivariate binary logistic regression were considered as independently associated statistically. The strength of association (odds ratio) was presented with a 95% confidence interval.

## 3. Results

### 3.1. Sociodemographic and Economic Description

A total of 384 participants were invited and fully participated in the study making a response rate of 100%. The majority (271, 76%) of the participants were males. The mean age and standard deviation of the participants were 32.75 years (±10.24 years). The largest proportion (203, 52.9%) of the participants were from a rural area, 234 (60.9%) were Muslim and 109 (28.4%) Orthodox, and 180 (46.9%) were single. Three hundred five (79.4%) of the participants had attended regular school. Regarding occupational status, 103 (26.8%) of the participants were students and 77 (20.1%) were farmers. The majority (245, 63.8%) of the participants were Oromo by ethnicity, followed by Amhara (60, 15.6%). The mean monthly family income and standard deviation were 1475.50 Ethiopian birr (±1432.70 Ethiopian birr), with minimum and maximum values of 50 and 10,000 Ethiopian birr, respectively. According to quartile income classification 108 (28.1%) patients had a monthly income greater than 1500 birr ($74.1), followed by 97 (25.3%) patients with monthly incomes of 650–1000 birr ($32.1–49.4). Regarding living condition 169 (944%) of the participants were living with their spouse and 149 (38.8%) were living with their families (Table 1).

#### 3.1.1. Description of Illness-Related Factors

Of the 384 participants, 154 (40.1%) were diagnosed with psychosis and 129 (33.6%) with depression. A total of 290 (75.5%) participants had history of verbal aggression and 93 (24.2%) of agitation or wandering in the street. The median (IQR) duration of mental illness was 3 (1.5–6.33) years, and the maximum and minimum durations of mental illness were 28 years and 1 month, respectively.

*Psychosocial Factors. *A large number of the participants believed that the cause of their mental illness was stress (n = 336, 87.5%) or thinking too much (n =280, 72.9%) and substance abuse (n = 186, 48.4%). Most (n=364, 94.8%) of the participants believed that symptoms of mental illness included anxiety, sleeplessness (n = 362, 94.3%), behavioral change (n = 329, 85.7%), and talking to oneself (n = 287, 74.7%). One hundred sixty-nine (44%) of the participants had a history of substance use or smoking or both within the last twelve months; 56 (33.13%) participants were using more than one substance.

Almost all (n=382, 99.5%) participants believe that mental illness is medically treatable. The majority (n=307, 79.9%) of the participants were receiving support from their families (n = 19, 4.95%), friends (n =14,3.7%), or a religious organization (n = 4, 1.04%): 243 (63.3%) were receiving moral support and 155 (40.4%) food support.

### 3.2. Medication-Related Factors

The median (IQR) duration of treatment in a health facility was 2 (1–6) years (maximum duration: 26 years; minimum duration: 1 month). Sixty-three (16.4%) participants were not taking their medication regularly: 54 (85.7%) had missed taking their regular medication 1–15 days per month; and 4 (6.34%), once within 3 days. Two-thirds (66.7%) of the participants had medication side effects, including sedation (n = 198, 51.9%), weight gain (n = 124, 32.3%), and tremor (n = 74, 19.3%). More than half the participants (n = 222, 57.8%) had used traditional medicines (herbal, spiritual, and others). As regards the number of visits to the psychiatric clinic, 301 (78.4%) participants had been to the clinic ≥5 times and 2 (0.5%) participants were attending their first visit.

#### 3.2.1. Magnitude of Perceived Stigma

The prevalence of perceived stigma of mental illness, i.e., agreement with at least one stigma item on the PDD scale, was 100%. However, the mean values revealed that 189 (49.2%) had low perceived stigma (score of < 2.5 points) and the rest (n = 195, 50.8%) had a high perceived stigma score (≥ 2.5 points). The reliability of the perceived devaluation-discrimination (PDD) scale was calculated and found to have Cronbach's alpha = 0.71.

The rate of perceived stigma of mental illness in this study was extensive. The respondents felt that the general public had a very negative attitude towards people with mental illness. The most frequently endorsed items of the PDD scale were as follows: almost all (365, 95%) participants agreed with the statement ‘‘*Most people think less of a person who has been in a mental hospital*” (PDD Item 7), 363 (94.5%) agreed with the statement “*Most young women would be reluctant to date a man who had been hospitalized for a serious mental illness*” (PDD Item 11), and 320 (83.3%) agreed with the statement ‘‘*Entering a mental hospital is a sign of personal failure*” (PDD Item 5). The highest level of disagreement was expressing the view that mentally ill people are neglected by the majority of the people, so that 353 (92.0%) participants disagreed with the statement “*Most people in my community would treat someone who has had mental illness just as they would treat any one*” (PDD Item 10), 350 (91.1%) participants disagreed with the statement “*Most people believe that a person who has been hospitalized for mental illness is just as intelligent as the average person*” (PDD Item 2), and 349 (90.9%) participants disagreed with “*A formerly mentally ill person would be accepted as a close friend by most people*” (PDD Item 1) (Table 2).

#### 3.2.2. Factors Associated with Perceived Stigma in Bivariate Analysis

Bivariate analysis was performed to get candidate variables for multivariate analysis. In bivariate analysis the variables were sociodemographic and economic related factors: being female, urban residency, Amhara ethnicity, monthly income, and getting family support (Table 3); among the psychosocial related factors in the bivariate analysis the variables were substance use, type of substance use (khat and alcohol), and perceived cause of mental illness (stress, thinking too much, God's order, and family history) (Table 4); and, from medication-related factors, in the bivariate analysis we found that duration of treatment, regularly taking medication, and having medication side effects were candidates for multivariate analysis at a* P* value <0.25 (Table 5).

#### 3.2.3. Independent Predictors of Perceived Stigma

Multiple logistic regression analysis was performed by using the backward stepwise (likelihood ratio) logistic regression method to know the independent predictors of perceived stigma by controlling for confounder variables. From the candidate variables, patients who had no substance use history were 0.6 times less likely to develop perceived stigma as compared to patients who had substance use history (AOR=0.6, 95% CI: 0.4–0.9), patients who had no medication side effect were 0.6 times less likely to develop perceived stigma as compared to patients who had medication side effect (AOR=0.6, 95% CI: 0.5–0.8), and patients having support from family (parents, relatives, spouse, and children) were 2.5 times more likely to develop perceived stigma as compared to patients who had no family support (AOR=2.5, 95% CI: 1.5–4.3) (Table 6).

## 4. Discussion

In this study, we found prevalence of low perceived stigma of 49.2% (mean PDD scale score < 2.5) and prevalence of high perceived stigma of 50.8% (mean PDD scale score ≥ 2.5). This finding is in line with a study performed in Pakistan that found that 49.09% of the participants had perceived stigma of mental illness [6]. It is higher than what was found in studies done in 16 countries of the world which showed that 13.5% of the overall sample had perceived stigma (22.1% in developing countries and 11.7% in developed ones) [10]. It is also lower than what was found in studies performed in Hong Kong (62.7%) [24], 13 European countries (71.6%) [25], and Southern Ghana (66.11%) [17].

Baseline responses on the PDD scale indicated that most study participants believed that people with current and former mental illness believed themselves to be devaluated. In the current study, 91% of the participants agreed or strongly agreed with the statement “*They will be seen as less intelligent*.” This is higher than the equivalent rates 71%, 57%, 67%, and 52.9% in studies performed in Southern Ghana, New York, New Jersey, and Hong Kong, respectively [17, 24, 26, 27]. Almost three-fourths (71%) of the study participants agreed with the statement that “*Employers will not hire persons with a former mental illness*.” This is slightly lower than the corresponding rates of 77% in Southern Ghana [17], 74% in New York [26], and 75.7% in Hong Kong [24].

Almost all (95%) the participants agreed with the statement that “*Most people would not accept a formerly mentally ill person as a close friend*.” This is higher than the corresponding rates of 80% in Ghana [17], 81% in New York [26], and 55.9% in Hong Kong [24]. A high number of participants (n = 349, 91%) agreed with the statement that* “Most young women would be reluctant to date a man who had a serious mental illness*.” This is higher than the corresponding rates of 58% in Ghana [17], 65% in New Jersey [27], 66% in New York [26], and 75.2% in Hong Kong [24]. A similar number of participants (n = 319, 83%) expressed the belief that “*Former persons with mental illness will be seen as less trustworthy*.” This is higher than the corresponding rates of 66% in Ghana [17], 53% in New Jersey [27], 69% in New York [26], and 60.6% in Hong Kong [24]. As regards the statement “*The opinions of mentally ill people will be taken less seriously*,” 347 (90%) participants expressed agreement. This is higher than the corresponding rates of 86%, 67%, and 66.4% in the studies performed in Southern Ghana, New York, and Hong Kong, respectively [17, 24, 26]. The discrepancy of all the above might be due to the sociocultural difference of the study participants.

From associated factors, patients who had support from family (parents, relatives, spouse, and children) were 2.5 times more likely to develop perceived stigma as compared to patients who had no family support. This is inconsistent with a study performed in the United States which found that perceived stigma was higher among those with lower socioeconomic status and those without any family members or friends who had used mental health services [15]. This might be due to over family care and attention which may affect the patient's social integration and needs further study.

In this study patients who had no substance use history were 0.6 times less likely to develop perceived stigma as compared to patients who had substance use history. This is supported by a narrative review of stigma in dual diagnosis patients with comorbid substance use disorder diagnosis experience to perceived stigma as compared to those with no comorbid diagnosis [28]. In a large survey conducted on the US general population respondents with AUD who had internalizing psychiatric comorbidity, as compared to those with no psychiatric comorbidity or externalizing comorbidity, had significantly higher levels of perceived alcohol stigma [29, 30].

From associated factors patients with no medication side effect were 0.6 time less likely to develop perceived stigma as compared to those with medication side effects. It might be due to the fact that patients with side effect may experience exclusion, rejection, blame, or devaluation that results from experience or reasonable anticipation of adverse effects of a medication by others.

The limitations of this study are as follows. The PDD scale was not validated in our setting. Also, there might be recall and social desirability bias, because the study was performed in a psychiatry clinic. The study was confined to patients who may not be representative of the general population. Additionally, some of the independent variables were assessed by single questions, for example, having medication side effects.

## 5. Conclusion

This study showed high prevalence of perceived stigma among persons with mental illness. Participants who were not receiving support from their families, those who had substance use history, and those with medication side effects had a high probability of having a high perceived stigma of mental illness. Therefore, special attention should be given to patients who have substance use, social support, and medication side effects.

## Figures and Tables

**Table 1:** Sociodemographic factors among adults with mental illness in JUSH, 2012.

Variable	Category	Frequency	
		Number	Percent
Sex	Male	271	70.6
	Female	113	29.4
Age	18–29	171	44.5
	30–39	115	29.9
	40–49	66	17.2
	50–59	22	5.7
	>60	10	2.6
Residence	Rural	203	52.9
	Urban	181	47.1
Religion	Muslim	234	60.9
	Orthodox	109	28.4
	Protestant	37	9.6
	Catholic	2	0.5
	Others	2	0.5
Marital status	Single	180	46.9
	Married	179	46.6
	Divorced	18	4.7
	Widowed	7	1.8
Educational status	Could not read and write	38	9.9
	Read and write (informal)	41	10.7
	Read and write (formal education)	305	79.4
Occupational status	Student	103	26.8
	Farmer	77	20.1
	Housewife	49	12.8
	Unemployed	45	11.7
	NGO employee	35	9.1
	Government employee	33	8.6
	Daily laborer	33	8.6
	Others	9	2.3
Monthly income	<650	90	23.4
	650–1000	97	25.3
	1000–1500	89	23.2
	>1500	108	28.1
Ethnicity	Oromo	245	63.8
	Amhara	60	15.6
	Gurage	25	6.5
	Tigrai	7	1.8
	Kefa	13	3.4
	Dawro	17	4.4
	Yem	14	3.6
	Others	3	0.8
Living with	Alone	38	9.9
	Family	149	38.8
	Relatives	27	7.0
	Spouse	169	44
	Friend	1	0.3

*Others* ^*E*^ includes Benjimaji and Wolayta, *Others*^*O*^ includes merchant and retired, *Others*^*M*^ includes divorced and separated, and *Others*^*R*^ includes Adventist and Jehovah's witness.

**Table 2:** Frequency of negative attitude of participants on the 12-item, 4-level Likert scale of the PDD scale at JUSH, 2012.

No.	The 12 items of perceived devaluation and discrimination (PDD) scale	Negative attitudes				
		Agree		Disagree		Total No.
		Freq	%	Freq	%	
1	Most people would willingly accept a person who has had mental illness as a close friend.	35	9.1	349	90.9	384
2	Most people believe that a person who has been hospitalized for mental illness is just as intelligent as the average person.	34	8.9	350	91.1	384
3	Most people believe that a person who has had mental illness is just as trust worthy as the average citizen.	65	16.9	319	83.1	384
4	Most people would accept a person who has fully recovered from mental illness as a teacher of young children in a public school.	45	11.7	339	88.3	384
5	Most people believe that entering a mental hospital is a sign of personal failure.	320	83.3	64	16.7	384
6	Most people will not hire a person who has had mental illness to take care of their children, even if he or she had been well for some time.	271	70.6	113	29.4	384
7	Most people think less of a person who has been in a mental hospital for treat.	365	95.0	19	5.0	384
8	Most employers will hire a person who has had mental illness if he or she is qualified for the job.	116	30.0	268	70.0	384
9	Most employers will pass over the application someone who has had mental illness in favor of another applicant.	279	72.7	105	27.3	384
10	Most people in my community would treat someone who has had mental illness just as they would treat any one.	31	8.0	353	92.0	384
11	Most young women would be reluctant to date a man who has been hospitalized for a serious mental illness.	363	94.5	21	5.5	384
12	Once they know a person was in a mental hospital for treatment, most people will take his/her opinions less seriously.	347	90.4	37	9.6	384

The scale was scored by adding scores on each item (after reverse scoring of the six items) and dividing by the number of items (12).

*Negative attitudes* represent the beliefs of the participants that they are devalued and discriminated against by others due to their illness.

**Table 3:** Association of sociodemographic and economic factors with perceived stigma in bivariate analysis among adults with mental illness in JUSH, 2012.

Variable	Category	Stigma status		COR	*P* value
		Low	High	(95% CI)	
Sex	Male	140 (51.7%)	131 (48.3%)	1	
	Female	49 (43.4%)	64 (56.6%)	1.4 (0.9–2.1)	0.14^*∗*^
Residency	Rural	106 (52.2%)	97 (47.8%)	1	
	Urban	83 (45.9%)	98 (54.1%)	1.3 (0.9–1.9)	0.21*∗*
Ethnicity	Oromo	126 (51.4%)	119 (48.6%)	1	
	Amhara	23 (38.3%)	37 (61.7%)	0.6 (0.3–1.0)	0.07*∗*
	Gurage	15 (60.0%)	10 (40.0%)	1.4 (0.6–3.3)	0.42
	Others^E^	25 (46.3%)	29 (53.7%)	0.8 (0.5–1.5)	0.50
Monthly income	<650	50 (53.6%)	40 (44.4%)	1	
	650–1000	50 (51.5%)	47 (48.5%)	0.9 (0.5–1.5)	0.58
	1000–1500	42 (47.2%)	47 (52.8%)	0.7 (0.4–1.3)	0.26
	>1500	47 (43.5%)	61 (56.5%)	0.6 (0.4–1.1)	0.10*∗*
Getting support from family (relatives, spouse, and children)	Yes	138 (45.0%)	169 (55.0%)	2.4 (1.4–4.1)	0.001^*∗∗*^
	No	51 (66.2%)	26 (33.8%)	1	
Social support other than family	Yes	7 (36.8%)	12 (63.2%)	1.7 (0.7–4.4)	0.27
	No	182 (49.9%)	183 (50.1%)	1	

*NB*. *∗*=*P* value <0.25.*∗∗*=*P* value<0.05.

*Others* ^*E*^ includes Tigrai, Kefa, Dawro, Yem, Benjimaji, and Wolayta.

**Table 4:** Association of psychosocial factors with perceived stigma among adults with mental illness in JUSH, 2012.

Variable		Category	Stigma of status		COR	*P* value
			Low	High	(95% CI)	
Substance use		No	99 (58.6%)	70 (41.4%)	0.5 (1.3–2.9)	0.001*∗∗*
		Yes	90 (41.9%)	125 (58.1%)	1	
Type of substance use	Khat use	No	73 (57.9%)	53 (42.1%)	0.6 (0.4–0.9)	0.008*∗∗*
		Yes	116 (45.0%)	142 (55.0%)	1	
	Alcohol	Yes	28 (65.1 %)	15 (34.9%)	0.5 (0.3–0.9)	0.03*∗∗*
		No	161 (47.2%)	180 (52.8%)	1	
Perceived cause of mental illness	Stress	Yes	172 (51.2%)	164 (48.8%)	0.5 (0.3–0.9)	0.04*∗∗*
		No	17 (35.4%)	31 (64.6%)	1	
	Thinking too much	Yes	146 (52.1%)	134 (47.9%)	0.6 (0.4–0.9)	0.06^*∗*^
		No	43 (41.3%)	61 (58.7%)	1	
	Substance abuse	Yes	90 (48.4%)	96 (51.6%)	1	
		No	99 (50.0%)	99 (50.0%)	0.9 (0.6–1.4)	0.75
	God's order	Yes	63 (55.3%)	51 (44.7%)	0.7 (0.5–1.4)	0.12^*∗*^
		No	126 (46.7%)	144 (53.3%)	1	
	Evil spirit	Yes	39 (46.4%)	45 (53.6%)	1	
		No	150 (50%)	150 (50%)	0.9 (0.5–1.4)	0.56
	Poverty	Yes	44 (51.2%)	42 (48.8%)	0.9 (0.6–1.5)	0.68
		No	145 (48.5%)	153 (51.3%)	1	
	Family history	Yes	18 (64.3%)	10 (35.7%)	0.5 (0.2–1.1)	0.10*∗*
		No	171(48.0%)	185 (52.0%)	1	
Perceived severity of mental illness		Mild	18 (58.1%)	13 (41.9%)	1	
		Moderate	36 (54.5%)	30 (45.5%)	0.9 (0.4–2.1)	0.75
		Severe	135 (47.0%)	152 (53.0%)	0.6 (0.3–1.4)	0.25

**Table 5:** Association of medication-related factors with perceived stigma among adults with mental illness in JUSH, 2012.

Variable	Category	Stigma status		COR	*P* value
		Low	High	(95% CI)	
Treatment duration	<1 year	52 (47.7%)	57 (52.3%)	1.5 (0.6–3.8)	0.37
	1–2 years	50 (58.1%)	36 (41.9%)	2.3 (1.0–5.9)	0.08*∗*
	2–5 years	43 (44.5%)	53 (55.2%)	1.3 (0.5–3.4)	0.52
	5–10 years	35 (50.7%)	34 (49.3%)	1.7 (0.7–4.4)	0.27
	>10 years	9 (37.5%)	15 (62.5%)	1	
Regularly taking the ordered medication	Yes	156 (48.6%)	165 (51.4%)	1.8 (1.2–2.7)	0.006*∗∗*
	No	33 (52.4%)	30 (47.6%)	1	
Medication side effects	Yes	140 (54.7%)	116 (45.3%)	1	
	No	79 (61.7%)	49 (38.3%)	0.5 (0.3–0.8)	0.003*∗∗*
Traditional treatment use history	Yes	106 (47.7%)	116 (52.3%)	1.2 (0.8–1.7)	0.50
	No	83 (51.2%)	79 (48.8%)	1	

*∗∗*  = *P* value < 0.05.*∗* = *P* value < 0.25.

**Table 6:** Multivariate analysis of factors associated with perceived stigma among adults with mental illness in JUSH, 2012.

Variables	Category	COR	AOR	*P* value
		(95% CI)	(95% CI)	
Getting support from family (relatives, spouse, children, and parents)	Yes	2.4 (1.4–4.1)	2.5 (1.5–4.3)	0.01*∗*
	No	1	1	
Substance use	Yes	1	1	
	No	0.5 (0.3–0.8)	0.6 (0.4–0.9)	0.001*∗*
Medication side effects	Yes	1	1	
	No	0.5 (0.3–0.8)	0.6 (0.5–0.8)	0.03^*∗*^

*∗* = *P* value <0.05.

## Data Availability

The data used to support the findings of this study are available from the corresponding author upon request.
